# Screening of native *Saccharomyces cerevisiae* strains from Chile for beer production

**DOI:** 10.3389/fmicb.2024.1345324

**Published:** 2024-02-09

**Authors:** Sandra Moreira-Ramos, Jorge Saavedra-Torrico, Camila G-Poblete, Liliana Godoy Olivares, Marcela Sangorrin, María Angélica Ganga

**Affiliations:** ^1^Departamento en Ciencia y Tecnología de los Alimentos, Facultad Tecnológica, Estación Central, Universidad de Santiago de Chile, Santiago, Chile; ^2^Escuela de Alimentos, Pontificia Universidad Católica de Valparaíso, Valparaíso, Chile; ^3^Departamento de Fruticultura y Enología, Facultad de Agronomía y Sistemas Naturales, Pontificia Universidad Católica de Chile, Santiago, Chile; ^4^Instituto de Investigación y Desarrollo en Ingeniería de Procesos, Biotecnología y Energías Alternativas (PROBIEN), Consejo Nacional de Investigaciones Científicas y Tecnológicas- Universidad Nacional del Comahue, Neuquén, Argentina

**Keywords:** native yeast, beer, flavor, *Saccharomyces cerevisiae*, artisan beer

## Abstract

**Introduction:**

Beer is one of the most consumed alcoholic drinks in the world, and this industry is a growing market that demands different properties to satisfy new consumers. The yeasts are used in different fermented beverages to contribute to new flavors. However, yeast strains used in the beer industry are limited so far, thus the diversity of flavors is very restricted. Therefore, the use of native yeast strains has been taking more importance with the purpose of conferring differentiated organoleptic properties to the product. Based on this observation the potentiality of native Saccharomyces cerevisiae strains obtained from different localities in Chile was researched.

**Methods:**

In this work was selected those strains that produced the highest ethanol concentration (nearly 6% v/v), consumed the highest amounts of sugars, and produced the lowest amounts of organic acids in the resulting beers. Finally, we did a beer tasting to select those strains that added different flavors to the final beer compared with a commercial strain used.

**Results and discussion:**

In this study, two native strains that produced fruity descriptors are described, which could be used in the future in brewing, craft or industrial production.

## 1 Introduction

Beer is one of the oldest and most consumed alcoholic drinks in the world (Colen and Swinnen, 2016). The elaboration of beer dates from ancient times, consisting of an infusion of germinated grain (wort) followed by fermentation in open containers (Unger, 2004). The transformation of the wort into beer was unknown until it was partially revealed in the 19th century when Pasteur showed that live microorganisms (yeasts) were responsible for the conversion of sugar into alcohol and CO_2_, in a process called alcoholic fermentation (Robbins, 2001). Due to the development and advances in microbiology, biotechnology, and the instrumentalization of the process, the quality and efficiency of beer elaboration has been improved (Hornsey, 2016). Modern beer elaboration consists of water, a starch source able to be saccharified and fermented, yeasts (*Saccharomyces* genus), responsible for alcoholic fermentation, and flavoring agents such as hops. In Chile, beer consumption has increased since 2012 (Euromonitor International Beer in Chile, 2016), reaching 45 L *per capita* annually; volumes that are surpassed at the Latin American level only by Brazil. Furthermore, men are usually the main consumers, while women are responsible for the dynamic growth of flavored beer (Euromonitor International Beer in Chile, 2016). Therefore, due to the new consumption trends of this product, it is important to increase the diversity in differentiated beers with new organoleptic properties.

Two factors determine the organoleptic characteristics of beer, one of them corresponds to the parameters used in fermentation and the other is the yeast strain chosen for wort fermentation [reviewed in Pires et al. (2014)]. Thus, brewing yeasts are a key component in flavor differentiation, because they are accountable for the production of compounds during fermentation that add flavor and aromas to the beer (White and Zainasheff, 2010; Pires et al., 2014), corresponding to metabolic intermediates or compounds resulting from the metabolism of wort, such as amino acids and carbohydrates. Brewing yeast strains are traditionally divided into two groups: top-fermenting strains used to produce beer types such as ale, stout, or porter, and bottom-fermenting strains used to produce lagers (Dengis et al., 1995). The difference between both types of yeast is that the yeasts from the top-fermenting group can ferment at higher temperatures than the bottom-fermenting strains, resulting in beers with “Fruity esters” flavors (Saerens et al., 2010). Nevertheless, because the diversity of yeasts in the market is very limited, there is difficulty in finding new approaches to producing differentiated beers without interfering with the balance of the final product or without adding undesirable aromas or flavors. Hence, the fermentative profile of top-fermenting yeasts results in a product with a standard aroma, flavor and body, encouraging the addition of other agents to the recipe of malt, hops, and yeast to obtain a differentiated product (Ibáñez, 2017). Moreover, the fermentation process is very accelerated, carrying out the loss of many compounds that grant aromas ester compounds such as ethyl hexanoate (banana flavor) and ethyl octanoate (flower aroma) and hexanoic acid (sweet aroma) (Olaniran et al., 2011). The resultant beers tend to have a very plain profile in flavors and tastes, decreasing the possibilities of producing beers with organoleptic conjugations that allow differentiation in the market (Fernandez-Robin et al., 2016). *Saccharomyces cerevisiae*, a top-fermenting yeast, is one of the most used yeasts in wine and beer production worldwide. Nowadays, in the beer industry, there are a few yeast species that are the most employed for fermentation, corresponding to commercial strains, but some artisanal producers employ spontaneous fermentation, with high variability and sometimes unpredictable results in terms of organoleptic properties. The study of the last type of beer production showed that there are different *S. cerevisiae* strains capable of wort fermentation (Pataro et al., 2000; Canonico et al., 2014; Larroque et al., 2021) and they are highly variable, showing different polymorphisms that could alter yeast metabolism, producing different profiles of secondary compounds which increase the diversity of flavor and aroma in the final product (Cabrera et al., 1988; Giudici et al., 1990; Pretorius, 2000; Fleet, 2003; Romano et al., 2003; Siesto et al., 2013; Cardoso et al., 2021). In addition, the different industries of fermentation beverages looking for new *S. cerevisiae* strains capable of fermenting more and adding different flavors in other alcoholic beverages such as wine (Rainieri and Pretorius, 2000; Suárez-Lepe and Morata, 2012; Ilieva et al., 2017; Costantini et al., 2019 Zhang et al., 2023), sparkling wine (Garofalo et al., 2018) and cider (Suárez-Valles et al., 2005; Kanwar and Keshani., 2016) and some studies have been carried out in beer (Lorca et al., 2022; Wauters et al., 2023). Our work group had previously isolated diverse native *S. cerevisiae* wine strains from different localities in Chile, and we determined that these strains could ferment wort. In this work, we look for new strains that promote diversity at the local brewing, something wanted after by new beer consumers.

## 2 Materials and methods

### 2.1 *Saccharomyces cerevisiae* strains

Beer elaboration was done with selected *S. cerevisiae* strains isolated previously from Chilean localities: III Region (29°54`28``S; 71°15`15`O), VII Region (35°25`36``S; 71°40`18``S), VIII Region (36°46`22``S; 73°03`47``O) and XIII Region (33°27`00``S; 70°40`00``O) (Table 1). These strains are part of the yeast collection of the Laboratorio de Biotecnología y Microbiología Aplicada (LAMAP) at the Universidad de Santiago. These strains were selected by fermentative capacity and technology projections (previously measured in terms of loss of weight, residual sugars, and final density) obtained from preliminary studies (data not shown). Loss of weight is a method used to estimate the fermentative power, which is the characteristic used to select one yeast over another. As a positive control, we used the commercial *S. cerevisiae* strain LalBrew (LalBrew Nottingham yeasts, Lallemand Brewing, Felixstowe, United Kingdom).

**Table 1:** Native *S. cerevisiae* strains used in preliminary study.

Locality (Region)	*S. cerevisiae* strain
Commercial	Lalbrew (LalBrew Nottingham yeasts, Lallemand Brewing, Felixstowe, United Kingdom)
III	III-A, III-B, III-C, III-D, III-E, III-F, III-G, III-H, III-I, III-J, III-K, III-L, III-M
VII	VII-A, VII-B, VII-C, VII-D, VII-E, VII-F, VII-G, VII-H, VII-I, VII-J, VII-K, VII-L, VII-M, VII-N,
VIII	VIII-A, VIII-B, VIII-C, VIII-D, VIII-E, VIII-F, VIII-G, VIII-H, VIII-I, VIII-J, VIII-K, VIII-L, VIII-M
XIII	XIII-A, XIII-B, XIII-C, XIII-D, XIII-E, XIII-F, XIII-G, XIII-H, XIII-I, XIII-J, XIII-K, XIII-L

### 2.2 Wort elaboration

We used the protocols for American Pale Ale beer preparation: To produce 20 L of wort, we mixed 6 kg of grain (Pale Ale) with 24 L of water previously heated at 70°C. Then, the mix was heated at 65°C for 90 min and, in the last 15 min, the mix was washed with 2 L of water. Then, the material was filtered. The filtered mixture was added to a new recipient, measuring the density. Then, the mixture was heated at 100°C, and 30 g of East Kent Golding hop was added. After 30 min, 20 g of East Kent Golding hop was also added, and the mixture was heated at 100°C for another 30 min. Once elaborated, the wort was cooled, filtered and kept at 4°C.

### 2.3 Microfermentation of the selected *Saccharomyces cerevisiae* strains (first stage)

Microfermentations of 10 mL were performed with selected *S. cerevisiae* strains (Table 1), *S. cerevisiae* LalBrew (positive assay) and a medium without yeast (negative assay). Each *S. cerevisiae* strain was grown in a medium that contained 4% maltose, 0.3% peptone and 0.5% yeast extract to increase cell biomass and then the yeasts were incubated for 36 h at 30°C with constant agitation (120 rpm). Finally, the strains were inoculated with a final concentration of 6.0 × 10^6^ cells/mL in a final volume of 10 mL (all trials were carried out in four replicates). The tubes containing the inoculated worts were placed in a chamber at 18 ± 1°C without agitation. It registered the chamber temperature twice daily (in the morning and the afternoon) for eight days. To control the fermentation process, the weight loss of each sample was tracked at different times. The result was expressed as CO_2_ production according to the following equation (expressed in g/L):

$$\frac{CO_{2}=\mathrm{initial}\;\mathrm{mass}-\mathrm{measured}\;\mathrm{mass}\;}{\mathrm{initial}\;\mathrm{volume}}\times 1000$$

Differences were assessed by ANOVA followed by mean separation using Fisher’s LSD test at 5% significance level. Then, we selected the best three yeasts for each Chilean locality that produced the highest CO_2_ production. The samples were maintained in a refrigerated chamber (4°C) for their maturation for 4 days.

### 2.4 Fermentations (second stage)

With the selected yeast strains in the first stage, we fermented 3 L of wort (elaborated in the first stage). We prepared three fermenters for each strain of three glass bottles (1 L), with a cork on the top, where an Airlock mechanism crossed the hole. The assays also included a positive control (*S. cerevisiae* LalBrew strain) and a negative control (without yeast). All the assays were done in triplicate. Each bottle was inoculated with 1 L of wort with the corresponding yeast strain, at a final concentration of 6.0 × 10^6^ cells/mL. The bottles were covered with the Airlock system and then they were stored in a fermentation chamber at 18 ± 1°C for 14 days. Then, the bottles were transferred to 4°C for 4 days to slow down the metabolism of yeasts. This protocol allowed the maturation of the beer, improving the fragrance and the precipitation of solids for better clarity. At the end of the maturation, the beer was pumped into glass bottles of 330 mL until the insoluble fraction. Then, it was gasified and inoculated with the same strain using initially at a final concentration of 6.0 × 10^6^ cells/mL. In addition, was added glucose into the beer at a final concentration of 8 g/L. Bottles were sealed with a manual cover, labeled, and stored at 18 ± 1°C for 7 days for a second fermentation, and then they were conserved in refrigeration until their analysis.

### 2.5 Physical and chemical analysis

All the beers were characterized by quantification of ethanol, citric acid, malic acid, succinic acid, lactic acid, acetic acid, maltose, glucose and glycerol. Those analysis were carried out in the Centro de Estudios en Ciencia y Tecnología de los Alimentos (CECTA, Universidad de Santiago) using liquid chromatography HPLC Shimadzu Prominence (Kyoto-Japan) using a column HPX-87H Aminex, 300 mm x 7.8 mm ion-exclusion column (BIO-RAD). The column was eluted at 55°C with 5 mM H_2_SO_4_ 5 mM at a flow rate of 0.6 mL/min in isocratic conditions. Two detectors were connected in series, where the organic acids were determined by Shimadzu SPD- M20A detector at 220 nm, while glucose, glycerol and ethanol were detected by Shimadzu refractive RID-10A index detector. The quantification was done using an external standard of each compound. The results integration and analysis were performed with LC Solution Software (Shimadzu). The calibration curves of each compound were using the following range of concentrations. Ethanol: 0.1 g/L – 98.36 g/L (*R*^2^ = 0.99977); citric acid: 0.1 g/L – 6.13 g/L (*R*^2^ = 0.99952), malic acid: 0.1 g/L – 6.12 g/L (*R*^2^ = 0.99840); succinic acid: 0.1 g/L – 5.03 g/L (*R*^2^ = 0.99941); acetic acid: 0.1 g/L – 5.20 g/L (*R*^2^ = 0.99901); lactic acid = 0.1 g/L – 5.47 g/L (*R*^2^ = 0.99981); maltose 0.1 g/L – 20.1 g/L (*R*^2^ = 0.99999); glucose = 2.0 g/L – 20.13 g/L (*R*^2^ = 0.99994) and glycerol 0.1 g/L − 10.04 g/L (*R*^2^ = 0.99930).

### 2.6 Sensory evaluation

We selected a group of ten beer tasters (three experts and seven trained tasters) all between the ages of 20 at 40 to carry out the sensory evaluation. The testing beer samples were those that had the highest scores in the physical and chemical analysis.

The beers were 1 h on ice and subsequently appropriately labeled with randomly generated numerical codes and delivered in tasting glasses (100 mL at 25°C). The sensory evaluations were carried out in the Food Building of the University of Santiago de Chile and were performed following the methodology indicated in ISO standards.

The attributes evaluated were odor-based olfactory sensations (through the nostril) and gustatory sensations (through the back of the throat). Sensory analysis was performed using a hedonic evaluation of each product based on a 5-point scale, where a score of 0 meant that the attribute was low, while a score of 5 indicated that the attribute was extremely high. The descriptors used in the trial corresponded to aroma (malt, hops, ester, and other aromas), flavors (malt, hops, balance and intensity of aftertaste), and flavor (alcohol, diacetyl/butter, DMS, ester/fruity, metallic, moldy, oxidized/aged, phenolic, solvent, acidic, bitter, vegetable and yeast). The average of all sensory panel evaluations per sample was used to obtain the final scores.

### 2.7 Statistical analysis

An ANOVA test was used to identify significant differences between the physical and chemical parameters determined after brewing. The sensory results were arranged into a dataset matrix (centered and scaled to unit variance) and subjected to Principal Component Analysis (PCA) and Partial Least Squares Discriminant Analysis (PLS-DA). These analyses were based on the nonlinear iterative partial least squares algorithm (NIPALS), which allows the analysis of a large number of highly correlated variables and ill-conditioned data, that is an incomplete rank matrix (dataset with more columns than rows). All models were validated by a full cross-validation routine, minimizing the prediction residual sum of squares statistics (PRESS) to avoid overfitting the model (Wold et al., 2001; Ferrer, 2007).

Furthermore, Correspondence Analysis (CA) was performed. This is a particular case of factor analysis to describe the relationships between nominal variables in a contingency table in a low-dimensional space, while simultaneously describing the relationships between the categories for each variable (Joaristi and Liza, 1999). The analysis shows how the tasters’ groups shape different clusters (homogenous groups) from similarities (or differences) of the sensory variables.

The analyses were validated by full cross-validation routines. All computes were performed with SIMCA-P+ 14® Umetrics AB, Sweden and IBM SPSS 25, IBM Corp.

## 3 Results

### 3.1 Yeast selection

**Figure 1:**
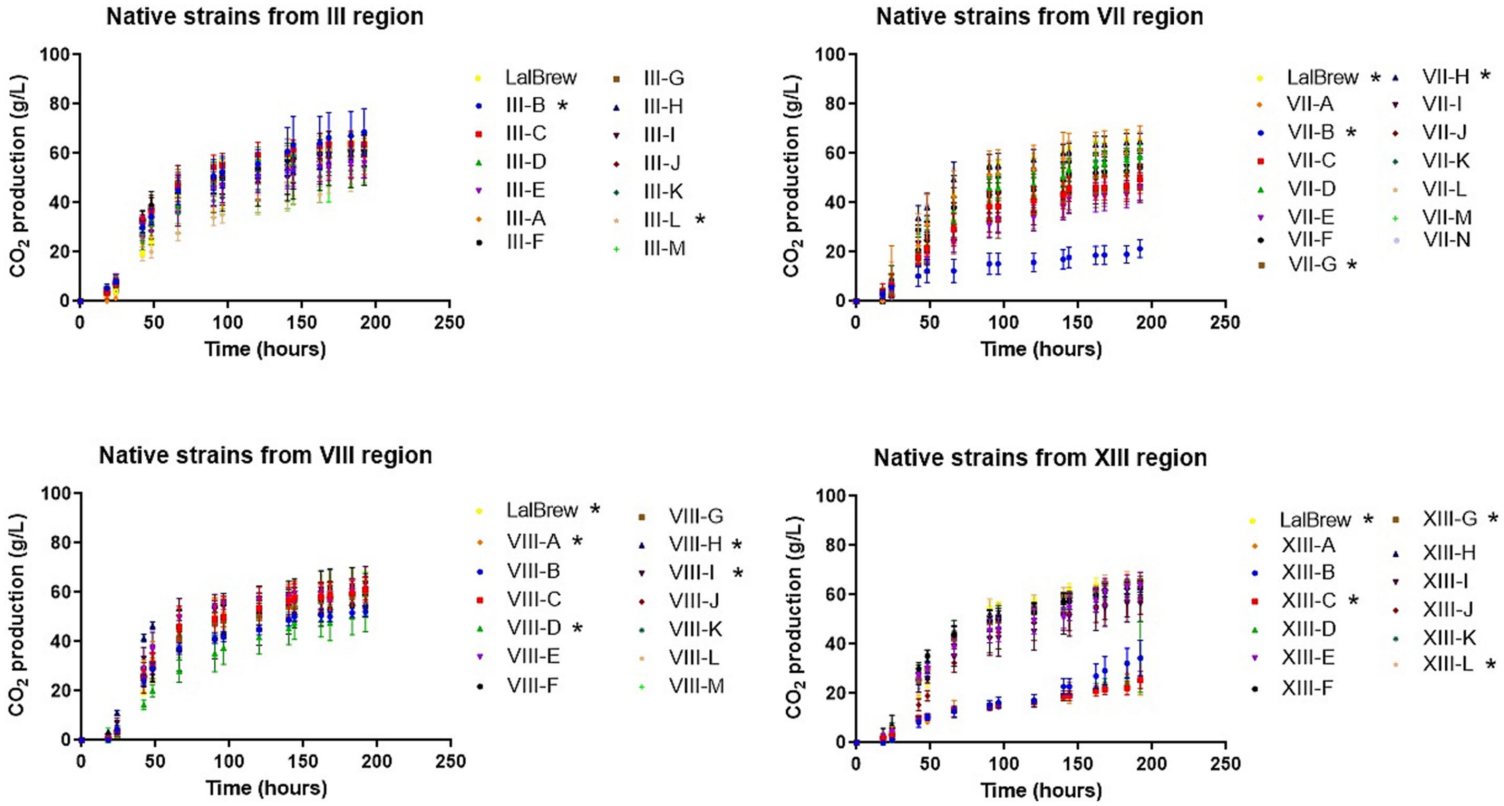
CO_2_ production in wort microfermentation of native and commercial *Saccharomyces cerevisiae* strain. CO_2_ production was estimated from data from weight loss. (*) shows the difference between assays at the end point of fermentation using ANOVA followed by mean separation using Fisher’s LSD test at 95% confidence level.

We carry out assays to determine the fermentative capacity of the set of yeast under study (Table 1). To estimate this parameter, we studied the CO_2_ production (Figure 1) (Vaughan-Martini and Martini, 1998). These data provide an estimated value of ethanol production, according to the following equation:

C6H12O6+2ADP+2Pi→2C2H6O+2CO2+2ATP+2H2O

In this equation, the production of one mole of CO_2_ is equivalent to the production of one mole of ethanol (C_2_H_6_O). In all regions, some yeasts have a fermentation capacity similar to LalBrew strain (positive control) (Figure 1). In III region the yeasts show an interesting fermentation potential, especially the III-B yeast which has, on average, a higher value than commercial yeast in terms of fermentation capacity. In the VII region, the VII-H strain had CO_2_ production similar to the control yeast (Figure 1). Conversely, the VII-B strain showed a tendecy to ferment slower, producing a lower CO2 concentration than the other yeast in the study. Selected strains from VIII region had the highest CO_2_ production on average in the first 90 h, compared to the positive control. At the end of the process, CO_2_ production was similar in the VIII-A, VIII-H, VIII-I, and LalBrew strains (Figure 1). For the XIII region, XIII-G, XIII-L and Lalbrew strains presented high production of CO_2_ at the end of the fermentation process (Figure 1). With the result obtained in these experiments, we selected three strains of each locality with the highest putative fermentative capacity (Table 2) to analyze their physicochemical properties in fermentations of 1 L. Table 3 shows the characterization of the beer samples elaborated where it is possible to observe and compare the concentration of succinic acid, citric acid, malic acid, acetic acid, maltose, glycerol and ethanol. We observed a relatively high concentration of succinic and acetic acid in all beers (>2 g/L). Strains that produced the lower concentration of acetic acid were III-C, IIID, VII-E, VII-H, VII-N, VIIIA, VII-H, VIII-I, XIII-H, XIII-G, and XIII-L. Beer elaborated with the LalBrew strain presented a concentration of 2.25 g/L. Citric and malic acid production showed similar values among strains from 0.38 to 0.69 g/L and around 0.57 to 0.97 g/L, respectively. In terms of sugar consumption which is directly correlated with the fermentative capacity of these strains, all the strains consumed the available glucose in the medium except the negative control (no inoculated). Maltose consumption was different among the strains, highlighting those strains that were able to metabolize more sugar than the others. Strains with the highest maltose consumption were: III-C, III-D, VII-H, VIII-A, VIII-H, VIII-I, XIII-G, XIII-H, XIII-G with averages values between 3.3 and 1.9 g/L. The LalBrew strain corresponding to 2.4 g/L. In the case of negative control, the maltose concentration was in average 67.1 g/L, the highest value among the samples. Also, we were interested in those yeast strains that produce an alcoholic content of around 6.0% v/v, like the commercial strain (positive control). Results showed that the VIII-H showed the highest alcohol content, similar to positive control (LalBrew strain). The rest of the strain showed average values between 6.20 at 4.14% (v/v). The native strain with the lowest alcohol concentration corresponds to the III-B (III Region), with a final concentration of 4.1% v/v. Regarding the pH measurements, all beers elaborated in this work presented a pH value within the national legal framework (4–4.5) (Decree N° 202, 1979). Thus, all analyzed strains are suitable for use in commercial beer production. Using the best beer obtained according to the physical and chemical parameters and discarding those that presented fragrances or flavors that were not characteristic were selected six yeasts (from highest to lowest): VII-H, VIII-A, XIII-H, XIII-L, VII-N and III-D.

**Table 2:** Selected *Saccharomyces cerevisiae* native strains according to the fermentative capacity.

Name	Locality (Region)
III-B	III
III-C	III
III-D	III
VII-E	VII
VII-H	VII
VII-N	VII
VIII-A	VIII
VIII-H	VIII
VIII-I	VIII
XIII-G	XIII
XIII-H	XIII
XIII-L	XIII

**Table 3:** Chemical analysis of beer.

Sample	Ethanol (%)	Succinic acid (g/L)	Citric acid (g/L)	Malic acid (g/L)	Acetic acid (g/L)	Maltose (g/L)	Glycerol (g/L)
Positive control (LalBrew)	6.03 ± 0.06^def^	3.50 ± 0.05^i^	0.42 ± 0.02^abcde^	0.75 ± 0.01^def^	2.25 ± 0.09^a^	2.42 ± 0.04^a^	2.47 ± 0.09^de^
Negative control	0.43 ± 0.346^a^	1.35 ± 0.24^a^	0.69 ± 0.05^f^	0.97 ± 0.09^g^	8.49 ± 0.92^b^	67.11 ± 2.37^e^	0.49 ± 0.28^a^
III-B	4.14 ± 1.126^b^	2.54 ± 0.14^bcd^	0.42 ± 0.01^bcde^	0.83 ± 0.03^f^	9.27 ± 1.83^b^	22.24 ± 1.21^d^	2.57 ± 0.26^def^
III-C	5.16 ± 0.41^cd^	2.64 ± 0.09^cde^	0.43 ± 0.01^bcde^	0.81 ± 0.13^ef^	2.96 ± 0.86^a^	3.29 ± 1.40^a^	3.03 ± 0.13^g^
III-D	5.48 ± 0.23^cdef^	2.69 ± 0.16^cde^	0.41 ± 0.01^abcd^	0.71 ± 0.01^cdef^	2.58 ± 0.35^a^	2.97 ± 0.76^a^	2.71 ± 0.02^ef^
VII-E	4.64 ± 0.83^bc^	2.45 ± 0.04^bc^	0.38 ± 0.05^a^	0.75 ± 0.09^def^	2.70 ± 0.98^a^	17.94 ± 1.06^c^	1.91 ± 0.24^c^
VII-H	5.96 ± 0.09^def^	2.94 ± 0.03^fg^	042 ± 0.02^abcde^	0.68 ± 0.02^bcd^	2.08 ± 0.03^a^	2.07 ± 0.07^a^	2.56 ± 0.13^def^
VII-N	5.34 ± 0.26^cdef^	2.38 ± 0.06^b^	0.39 ± 0.01^ab^	0.69 ± 0.01^abcde^	2.17 ± 0.13^a^	13.15 ± 1.86^b^	2.31 ± 0.09^d^
VIII-A	5.23 ± 0.49^cde^	3.09 ± 0.02^gh^	0.40 ± 0.01^abc^	0.69 ± 0.08^abcde^	2.84 ± 0.86^a^	2.19 ± 0.05^a^	2.48 ± 0.07^de^
VIII-H	6.06 ± 0.21^cef^	3.71 ± 0.12^j^	0.44 ± 0.02^de^	0.57 ± 0.07^a^	2.01 ± 0.02^a^	1.90 ± 0.06^a^	2.99 ± 0.02^g^
VIII-I	6.20 ± 0.41^f^	2.78 ± 0.09^ef^	0.43 ± 0.01^cde^	0.69 ± 0.02^abcde^	2.06 ± 0.04^a^	1.98 ± 0.25^a^	2.80 ± 0.06^fg^
XIII-G	5.55 ± 0.07^def^	3.77 ± 0.12^j^	0.43 ± 0.01^bcde^	0.59 ± 0.16^ab^	2.13 ± 0.06^a^	2.33 ± 0.07^a^	1.58 ± 0.07^b^
XIII-H	5.32 ± 0.69^de^	2.97 ± 0.20^fg^	0.42 ± 0.01^bce^	0.61 ± 0.09^abc^	2.65 ± 0.59^a^	2.37 ± 0.04^a^	2.65 ± 0.34^ef^
XIII-L	5.61 ± 0.12^def^	3.25 ± 0.02^h^	0.45 ± 0.016^e^	0.71 ± 0.02^bcde^	2.33 ± 0.36^a^	2.39 ± 0.04^a^	2.81 ± 0.02^fg^

Multiple Comparisons determined by Fisher’s LSD Methods at 95% confidence level. Different upper-case letters in the same column indicate significant differences (*p* < 0.05) between samples.

### 3.2 Sensory analysis

**Figure 2:**
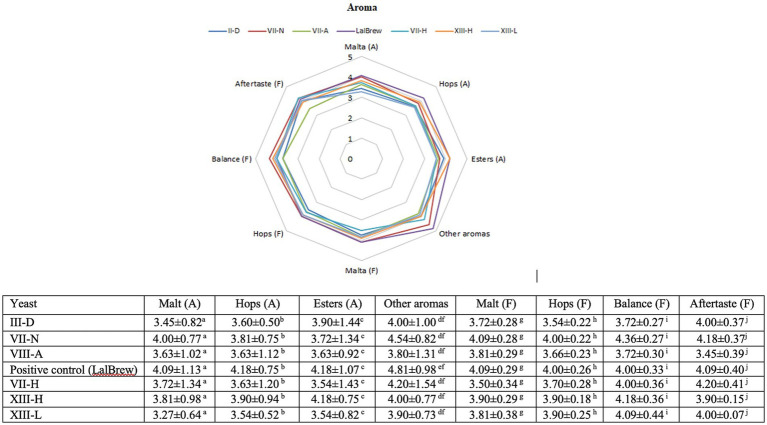
Sensory analysis of the finished beers. Spider plots for average aromas determined by the judges. Beer obtained by: III-D (dark blue), VII-N (red), VIII-A (green), LalBrew/commercial (purple), VII-H (light blue), XIII-H (orange), and XIII-L (Light Blue). The table was done using Multiple Comparisons determined by Fisher’s LSD Methods at 95% confidence level. Different upper-case letters in the same column indicate significant differences (*p* < 0.05) between samples.

The results of the sensory evaluation are presented in Figure 2. The aroma associated with malt, hops and esters did not present statistically significant differences between the beers fermented with native yeasts versus the control yeast. Only the attribute “others aroma” showed some difference between commercial yeast and native yeasts (Figure 2).

**Figure 3:**
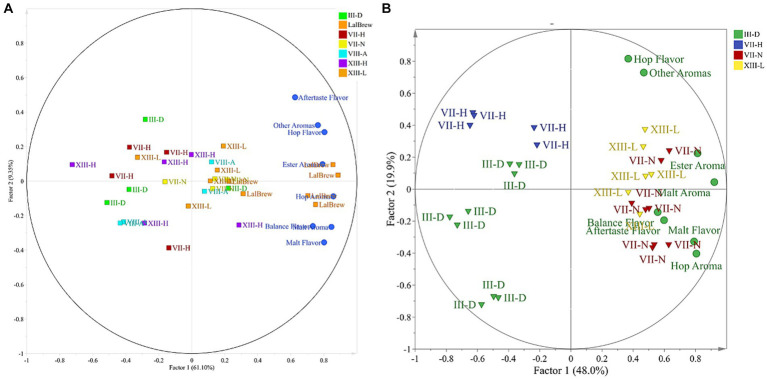
Biplot of loadings and scores of the projection for beers analyzed by sensory test. **(A)** Considering commercial yeast strain. **(B)** Regardless to the commercial yeast strain.

**Figure 4:**
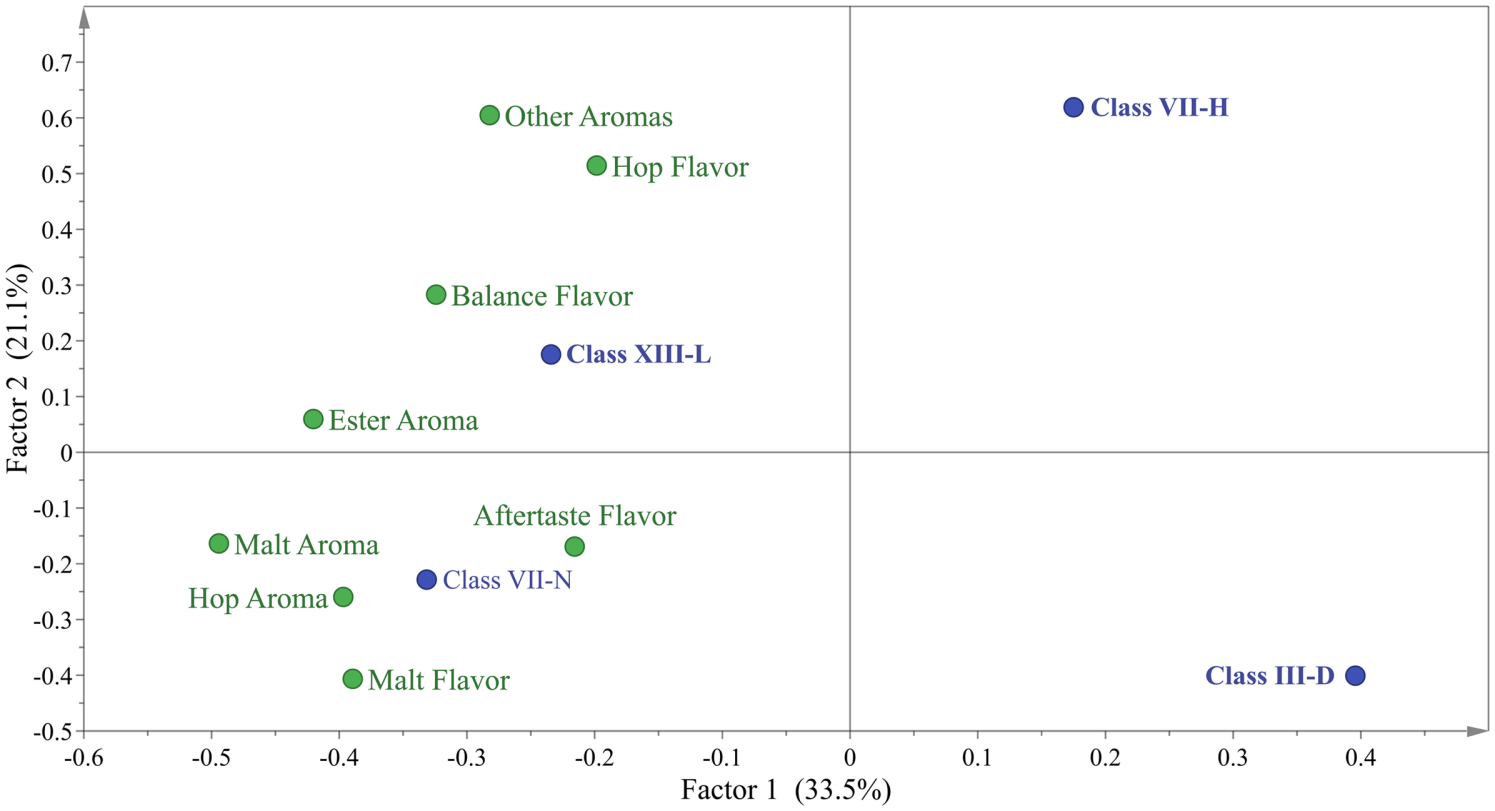
Discriminant projection plot for beers analyzed by sensory test.

PCA was applied to the sensory test as an exploratory multivariate statistical technique to differentiate the samples. However, 3 beer samples presented great variability in the described profiles, so they were excluded to improve the analysis. Based on this, a new PCA was carried out with the 4 remaining beer samples (Figure 3). The sensory dataset extracted two factors that explained 70.45% of the total variability. In the case of Factor 1, t1 (61.10% of the variance) sorted the samples from left to right according to the sensory score given by the testers panel. Factor 2, t2 (9.35% of the variance) sorted the samples related to the spectra of the different variables tested. By exploring the internal order of the scores (beer projection) and considering the high variability nature of the sensory data. The LalBrew beer group is to the right of the projection, while the other beers appear on the center-left side (Figure 3A). When was eliminate the commercial strain from this analysis, it is possible to observe that it maintains the dispersion in the native yeasts except for the XIII-L strain showing a relationship between ester aroma, hop aroma, aftertaste flavor, and balance flavor (Figure 3B), similar to what was obtained with the commercial strain observed in Figure 3A. Considering the information in Figure 3B, the dataset was subjected to Discriminant Analysis by PLS-DA. The PLS-DA analysis extracted 3 components that explained 54.6% of the total variability in matrix Y. In Figure 4, it can be observed again that the XIII-L yeast correlates with the qualities of balanced flavor, ester aroma, and hop flavor. In the case of beers with VII-H yeast, their characteristics are contrary to these qualities, that is, very low correlation with hop flavor, balanced flavor, ester aromas, and other aromas. In the case of beers with strain VII-N, there is a high correlation with malt flavor, aftertaste flavor, hop aroma, and malt aroma, while for yeast III-D the correlation was very low with these same attributes. Concerning the additional descriptors detected by the sensory panel, the dataset was subjected to correspondence analysis regarding the presence or absence of an attribute. This analysis showed that all beer done with the yeast native had a higher relationship with the ester aroma.

## 4 Discussion

The study of the fermentative capacity of the strains analyzed showed clear differences between them. The strains that on average presented the lowest fermentative capacity (quantified as CO_2_ loss) were strains VII-B, XIII-C, XIII-A, contrary to strains III-B, VII-H, VIII-A, VII-H, VII-I, and XIII-G (Figure 1). These latter strains had similar behavior to the commercial LalBrew strain used as a positive control of the fermentation process. To reduce the number of samples, the yeasts with the highest fermentative capacity were selected, obtaining at least 3 from each region. In this way, a final universe of 12 beers with different strains of *S. cerevisiae* was obtained, on which physicochemical analyses were carried out. The beer produced with the commercial strain reached an ethanol concentration average of 6.0% w/v, while only 2 native yeast strains reached a concentration average equal to or greater than 6.0%. In the case of the LalBrew strain, it can produce beers between 4.6 to 6.3% depending on the wort used (Paguet et al., 2024), values that are within what was obtained in this study. When analyzing the glucose concentration of the beers produced, it is possible to observe that, in all the samples inoculated with yeast, the glucose concentration was not detected in the final product. The alcohol concentration is related to the sugar that yeast metabolizes to ethanol. Yeasts can activate other metabolic pathways that would allow the production of compounds other than ethanol (Lambrechts and Pretorius, 2000). Among these compounds, glycerol is one of them, which plays an important role in maintaining intracellular redox balance under anaerobic conditions (Van Dijken and Scheffers, 1986). In addition, glycerol provides positive aromatic qualities to beers, giving fullness and body (Cardoso et al., 2021). In this case, all beers obtained an average glycerol concentration between 1.58 to 3.0 g/L (without considering the non-inoculated sample). The values obtained were similar to those of Cardoso et al. (2021) when testing with different commercial yeasts. In that case, these authors obtained beers with glycerol concentrations between 1.17 to 2.4 g/L. In the beers produced, it was observed that the negative control (test without inoculation) obtained a concentration of 67.1 g/L of maltose, followed by the yeasts III-B, VII-E and VII-N with an average of 22.2, 17.9 and 13.1 g/L, respectively. The rest of the yeasts consumed the maltose, leaving an average value of less than 3.3 g/L, while the commercial yeast left only 2.4 g/L of maltose in the product.

For its part, analyses of organic acids were also carried out on the beers produced, this is because their presence can influence the aromatic profile of the beers, also affecting the pH. In addition, organic acids could contribute to beer’s salty and sour taste, but in high concentrations, they would negatively affect the product (Cardoso et al., 2021). In our work, the presence of lactic acid was detected only in that test that was not inoculated with yeast, which could be due to contamination with lactic acid bacteria (0.3 ± 0.04 g/L). On the other hand, the beer from yeast III-B showed the highest concentration of acetic acid at 9.2 g/L, while the rest were at values between 2.9 to 2.0 g/L (including the positive control). In the case of wine yeasts, there is a relationship between the concentration of acetic acid and osmotic stress. A greater production of glycerol and acetic acid would be related to stress tolerance (Novo et al., 2014), contrary to what was observed in our study since the product of strain III-B was the one that on average had the lowest concentration of ethanol (4.1% v/v). A high concentration of acetic acid (about 300 ppm) gives a vinegary fragrance which is unwanted in beer (Mizuno et al., 2006). Because of this, this beer was not tested. On the other hand, some studies indicate that there would be an increase in the concentration of succinic acid in fermented beverages, however, the beers produced in this work had a low concentration of this organic acid, which would translate into having less salty and bitter beers (Xu et al., 2018). The differences in organoleptic properties of the beer are due to the production of second metabolites, specifically volatile compounds, such as ester, alcohols, aldehydes, dicarbonyls, short-chain fatty acids, phenolic compounds and terpenes, among others (Styger et al., 2011) which are generated from *de novo* or by metabolizing molecules present in the starting material (hop, malt, etc.) (Lambrechts and Pretorius, 2000; Moreno-Arribas and Polo, 2009). The commercial strain delivered beers with organoleptic qualities closely related to the raw material (hop aroma, malt aroma) (Figure 3A). These results are expected because this strain has been domesticated for centuries, and this is correlated with a high capacity of attenuation that allows the sense of hop and wort flavors and aromas (Bell et al., 2001; Paguet et al., 2024). Furthermore, Dietz et al. (2020) detected the presence of citronellol in some beer could be derived from a *de novo* synthesis induced by the yeast used in the fermentation. The production of these compounds, and their amount is particular to each strain because the secondary metabolism is influenced by their adaptation to the environment where they were isolated. The sensory test showed the native strains have no difference between them (Figure 2). However, the Figure 3 showed the XIII-L strain had a high relationship with ester descriptors. Esters contribute to floral and fruity characteristics in beer, and these molecules are generated by the esterification of alcohol and acids at low pH (Saerens et al., 2010). Among esters produced by *S. cerevisiae* are ethyl acetate, isoamyl acetate, 2-methyl butyl acetate and phenyl ethyl acetate, described as banana, apple, fruit, aromatic sweetness and the most common ester is ethyl acetate, produced from ethanol (Saerens et al., 2010). It is known that several genes have been associated with the production and degradation of esters. However, the mechanisms of ester production are poorly understood. The commercial yeast showed a high relationship with the aromas of hops and malt (Figure 3A), aromas typical of the raw material (Paguet et al., 2024). This yeast is characterized by being a neutral yeast, that is, it does not produce other aromas that alter those of hops and malt. To improve the characterization of beers brewed with native *S. cerevisiae* by PCA, the commercial strain was eliminated (Figure 3B). This allowed us to observe a greater distance between the beers produced with the native strains, clearly showing the groups of III-D and VII-H that are distant from XIII-L and VII-N, the latter closely related to ester aromas, malt aroma, malt flavor, balance flavor, aftertaste flavor, and hop aroma. The III-B strain produced a beer with a lower alcohol content in average compared to the control strain. This would be a good quality to consider for making low-alcohol beers. Furthermore, this native strain consumed all the glucose in the must and produced a glycerol concentration similar to the commercial strain (Table 3). The beer made with the XIII-L strain shows a relationship with ester, the XIII-H with oxidized aroma, III-D with phenolic aroma, VII-N with bitter and oxidized sensation (Figure 4). While vegetables and yeast aromas are related to VIII-A strain. In the case of LalBrew beer was characterized by alcohol, moldy and ester aroma. In our study the natives’ yeast gave different aromas of the raw material, similar results were obtained by Larroque et al. (2021) who showed that the native’s *S. cerevisiae* showed better sensory characteristics than the commercial strain used. It is interesting to delve into the production of aromas of the VII-H and XIII-L strains and in yeast with lower ethanol production. Yeast selection is crucial to produce new types of beer thus allowing the differentiation of products between regions, which can further the creation of new craft breweries.

## Data availability statement

The original contributions presented in the study are included in the article/supplementary material, further inquiries can be directed to the corresponding author.

## Author contributions

SM-R: Writing – original draft, Formal analysis, Methodology. JS-T: Writing – original draft, Formal analysis, Writing – review & editing. CG-P: Formal analysis, Writing – original draft, Writing – review & editing, Funding acquisition. LGO: Formal analysis, Writing – original draft, Writing – review & editing, Funding acquisition, Supervision. MS: Writing – original draft, Writing – review & editing, Methodology. MG: Funding acquisition, Supervision, Writing – original draft, Writing – review & editing, Conceptualization, Investigation, Project administration, Resources.
